# Impact of specialization in gynecology and obstetrics departments on pregnant women’s choice of maternity institutions

**DOI:** 10.1186/2191-1991-3-31

**Published:** 2013-12-23

**Authors:** Yoshimi Adachi, Hiroyasu Iso, Junyi Shen, Kanami Ban, On Fukui, Hiroyuki Hashimoto, Takako Nakashima, Kenichiro Morishige, Tatuyoshi Saijo

**Affiliations:** 1 Public Health, Social and Environmental Medicine, Graduate School of Medicine, Osaka University, 2-2 Yamadaoka, Suita, 565-0871, Osaka, Japan; 2 Research Institute for Economics and Business Administration, Kobe University, 2-1 Rokkodai, Kobe, 657-8501, Hyogo, Japan; 3 Graduate School of Economics, Osaka University, 1-7 Machikaneyama, Toyonaka, 560-0043, Osaka, Japan; 4 Department of Obstetrics and Gynecology, Izumisano City Hospital, 2-23 Rinku Ourai Kita, Izumisanoshi, 598-8577, Osaka, Japan; 5 Department of Obstetrics and Gynecology, Kaizuka City Hospital, 3-10-20 Hori, Kaizukashi, 597-0015, Osaka, Japan; 6 Faculty of Service Industries, University of Marketing and Distribution Sciences, 3-1 Gakuen-Nishimachi, Nishi-ku, Kobe, 651-2188, Hyogo, Japan; 7 Graduate School of Medicine, Gifu University, 1-1 Yanagido, Gifu, 651-2188, Japan; 8 School of Management, Kochi University of Technology, Kami, 782-8502 Kochi, Japan

**Keywords:** Specialization, Gynecology and obstetrics departments, Maternity facilities, Before and after analysis

## Abstract

In April 2008, specialization in gynecology and obstetrics departments was introduced in the Sennan area of Osaka prefecture in Japan that aimed at solving the problems of regional provisions of obstetrics services (e.g., shortage of obstetricians, overworking of obstetricians, and provision of specialist maternity services for high-risk pregnancies). Under this specialization, the gynecology and obstetrics departments in two city hospitals were combined and reconstructed into two centers, i.e., the gynecological care center in Kaizuka City Hospital and the prenatal care center in Izumisano City Hospital. This paper investigates to what extent and how this specialization affected pregnant women’s choices of the prenatal care center and other maternity institutions. We used birth certificate data of 15,927 newborns from the Sennan area between April 1, 2007 and March 30, 2010, for Before and After Analysis to examine changes in pregnant women’s choices of maternity institutions before and after the specialization was instituted. Our results indicated that this specialization scheme was, to some extent, successful on the basis of providing maternity services for high-risk pregnancies at the prenatal care center (i.e., Izumisano City Hospital) and having created a positive effect by pregnant women to other facilities in the nearby area.

## Background

In the Sennan area (i.e., Southern area) of Osaka Prefecture in Japan, gynecology and obstetrics departments of two small- to- medium-sized hospitals had been closed, and the shortage of physicians in gynecology and obstetrics departments had become a serious problem. The work burdens on remaining physicians in that area had been increasing (i.e., the number of patients seen, the number of shifts on call, and the number of hours worked), and they found it increasingly difficult to provide highly-specialized medical care.

In April 2008, a specialization in gynecology and obstetrics departments was conducted in the Sennan area. Under this specialization, the gynecology and obstetrics departments in Kaizuka City Hospital and Izumisano City Hospital were merged into the Mother and Child Medical Center. Then, this center was reconstructed into two new sub-centers. One was called the gynecological care center, which is operated by Kaizuka City Hospital. The other center, called the prenatal care center, is operated by Izumisano City Hospital because this hospital has the neonatal intensive care unit and is located close to the accident and emergency center. Because of this specialization, the pregnant women who were examined in Kaizuka City Hospital would move to Izumisano City Hospital when risk of complication in birth became high or when they started having contractions, while pregnant women who had been examined in Izumisano City Hospital would remain at the same hospital.

This specialization in gynecology and obstetrics departments which aimed at solving the problems of regional provisions of obstetrics services (e.g., shortage of obstetricians and maternity services for high-risk pregnancies, and over working of obstetricians) has three main advantages. First, each hospital can concentrate on providing the core care services within its specialization by completely utilizing its existing facilities. Second, this specialization enables the provision of more advanced medical services. Third, since the physicians from Kaizuka City Hospital are also involved in after-hour care at Izumisano City Hospital, the problem of overworked gynecologists and obstetricians in both hospitals will be mitigated to some extent by the adoption of a two-physicians-on-duty system. In Japan, because of the shortage of gynecologists and obstetricians, some hospitals often closed gynecology and obstetrics departments in one hospital to maintain those in another hospital.

The specialization undertaken in the Sennan area can be regarded as a new approach in Japan because it would prevent the closure of gynecology and obstetrics departments in either hospital. This specialization enables the prenatal care center of one hospital to provide more advanced maternity services for high-risk pregnancies, and the gynecology center of the other hospital to provide more advanced gynecological services. Meanwhile, this specialization is considered to have a possible positive effect on other maternity facilities since it allows them the opportunity to attract low-risk pregnancies. In this evaluation of the specialization, we investigated the following research questions.

1) Was the specialization undertaken by gynecology and obstetrics departments a valid approach to improve the regional provisions of obstetrical service in the Sennan area?

2) Did the specialization of gynecology and obstetrics departments in Kaizuka City Hospital and Izumisano City Hospital affect pregnant women’s choices? More specifically, we investigated whether the specialization would bring an effect on pregnant women choosing other maternity facilities, either positive or negative.

The remainder of the paper is organized as follows. In the next session, we provide the detailed reviews on related studies. Section 3 presents the methodology issues. Results and discussions are given in Sections 4 and 5, respectively. Finally, Section 6 draws the conclusions.

### Related studies

To the best of our knowledge, except for the above mentioned specialization such a specialization that prevents the closure of any gynecology and obstetrics department in either hospital has not been conducted in Japan and other countries. Therefore, we are unable to find many studies related to ours except for two studies. One study [1] applied a hypothetical choice experiment method through a mail survey for women who had delivered in Kaizuka City Hospital between 2003 and 2007, investigated the kinds of factors affecting pregnant women’s choice of maternity facilities and created a database for further studies. On the basis of the data collected in [1], the authors of [2] implemented Cost-Benefit Analysis of the specialization for gynecology and obstetrics departments in Kaizuka City Hospital and Izumisano City Hospital. By estimating consumers’ benefits and calculating producers’ surplus and costs, they reported that the benefit-cost ratio was estimated at 1.367 under a basic scenario, indicating that the specialization can generate a net benefit.

Several related studies have discussed the factors behind patients’ choice of hospital. One study examined the impact of waiting and travel times on the choice of cataract patients in the United Kingdom [3]. That study found that travel time had a stronger effect on the choice of hospital than did waiting time [4] and [5] reported that patients responded to not only convenience but also quality and reputation when choosing fertility clinics for assisted reproductive therapies.

In addition, several studies examined issues regarding changes in hospital organization, such as mergers and closures. Among these studies, three examined the effects of hospital mergers in terms of cost and management [6] investigated the outcomes of hospital financial performances before and after physician integration strategies were executed between hospitals. The result indicated that a hospital merger led to cost savings and financial benefits. However, the robustness of financial benefits in terms of hospital size and organization has since been debated [7] conducted a six-year longitudinal assessment of change before and after a merger. In that study, the short-term impact of a merger was generally modest, but the impact of mergers between similarly-sized hospitals reflected greater efficiency than mergers between dissimilar-sized hospitals [8] analyzed the costs and prices of approximately 3,500 short-term general hospitals from 1986 to 1994. The hospital mergers produced average cost savings of approximately 5%. These savings were generally greater for mergers between similarly-sized hospitals, and the post-merger price reductions were smaller in less competitive markets than in competitive ones. These studies showed that depending upon their scale, hospital mergers led to different levels of efficiency. Other studies asserted the need to understand not only the primary motivation of hospitals but also the overall status of hospitals prior to a merger [9] found it that a hospital merger would lead to different levels of efficiency depending upon hospital location, because some hospitals were pressured by their local competitors to increase management efficiency [10] investigated the pre-exit characteristics of hospital mergers and closures using an 18-year national data set that spanned the wave of closures in the 1980s and mergers in the 1990s. That study suggested that weak productivity was a strong determinant for hospital closures, while competitive pressure was influential in the decision to consolidate [11] examined a possible horizontal merger between urban hospitals and suggested that the merger would be effective in hospital ownership, governance, and market structure.

## Methods

We collected birth certificate data from the Izumisano and Kishiwada Health Centers between April 1, 2007 and March 30, 2010, and examined pregnant women’s choices and regional provisions of obstetrical services after the specialization. These public health centers are in charge of the registrations of all newborn babies in the Sennan area. Therefore, our sample of births can be viewed as a representative sample of the investigated area.

**Figure 1:**
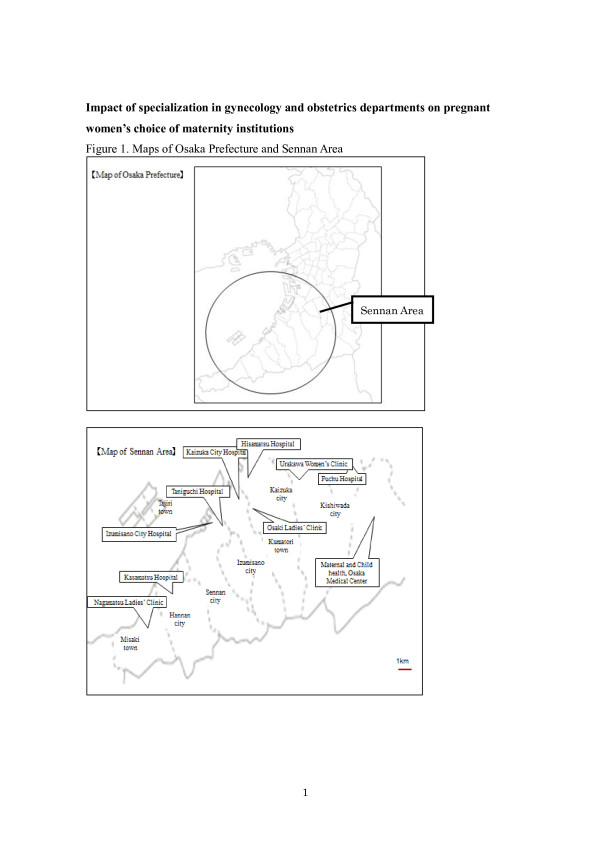
Maps of Osaka prefecture and Sennan area.

We examined the number and proportion of total births, low birth weight births, and premature births in all regions and the three cities involved in the specialization (Izumisano, Kaizuka, and Kumatori cities) by maternity facility among pregnant women in the Sennan area during the pre-specialization period, the first year after specialization, and the second year after specialization. Since the specialization in gynecology and obstetrics departments started on April 1, 2008, the period from April 1, 2007 to March 31, 2008, refers to the pre-specialization period. Accordingly, the first and second year after the specialization denote the periods between April 1, 2008 to March 31, 2009, and April 1, 2009 to March 31, 2010, respectively. In addition, a low birth weight was defined as the child’s birth weight being lower than 2,500 grams, while a premature birth referred to a birth with a gestational age lower than 37 weeks. A map of cities/towns and main maternity facilities in the Sennan area is provided in Figure 1 to provide an overview of the Sennan area and help readers understand Table 1 and Table 2.

**Table 1:** Total births, low birth weight and premature births registered in all regions of the Sennan area

	**Total births**			**Births with low birth weight**						**Premature births**					
	**Pre-specialization**	**First year after specialization**	**Second year after specialization**	**Pre-specialization**		**First year after specialization**		**Second year after specialization**		**Pre-specialization**		**First year after specialization**		**Second year after specialization**	
**Izumisano city**															
Izumisano City Hospital	647	844	950	101	(16)	123	(15)	162	(17)	41	(6)	73	(9)	100	(11)
Taniguchi Hospital	1,032	986	1,075	66	(6)	63	(6)	89	(8)	22	(2)	19	(2)	39	(4)
**Kumatori city**															
No Hospital or Clinic	0	0	0		(0)		(0)		(0)		(0)		(0)		(0)
**Kaisuka city**															
Kaisuka City Hospital	536	0	0	61	(11)	0	(0)	0	(0)	29	(5)	0	(0)	0	(0)
Osaki Ladies’ Clinic	490	510	516	37	(8)	25	(5)	39	(8)	10	(2)	12	(2)	18	(3)
Hisamatsu Hospital	433	524	454	25	(6)	28	(5)	25	(6)	14	(3)	18	(3)	10	(2)
**North of Kishiwada city**															
Fuchu Hospital	204	285	304	23	(11)	26	(9)	27	(9)	8	(4)	11	(4)	8	(3)
Urakawa Women’s Clinic	178	215	233	9	(5)	6	(3)	16	(7)	6	(3)	2	(1)	5	(2)
Maternal and Child Health, Osaka Medical Center	144	175	167	43	(30)	61	(35)	59	(35)	35	(24)	48	(27)	42	(25)
Oigi Ladies’ Clinic	92	122	97	2	(2)	7	(6)	10	(10)	2	(2)	4	(3)	6	(6)
Wada Tokushukai Coast Hospital	83	93	113	3	(4)	3	(3)	3	(3)	1	(1)	3	(3)	1	(1)
Izumiotsu City Hospital	61	90	89	5	(8)	8	(9)	11	(12)	4	(7)	5	(6)	4	(4)
Izumi City Hospital	18	12	0	5	(28)	0	(0)	0	(0)	0	(0)	0	(0)	0	(0)
Southern Sennan Area															
Kasamatsu Women’s Clinic	262	243	250	17	(6)	22	(9)	21	(8)	4	(2)	5	(2)	2	(1)
Nagamatsu Ladies’ Clinic	252	263	178	19	(8)	26	(10)	15	(8)	26	(10)	20	(8)	5	(3)
**Facilities outside the Sennan area**															
No Facility Name Available	14	101	21	4	(29)	12	(12)	2	(10)	3	(21)	8	(8)	0	(0)
**Total**	5,376	5,324	5,227	534		515		558		289		301		287	

*In parentheses, the percent proportions among total births were presented.

**Table 2:** Total births, low birth weight and premature births registered in Izumisano, Kaizuka, and Kumatori

	**Total births**						**Births with low birth weight**						**Premature births**					
	**Pre-specialization**		**First year after specialization**		**Second year after specialization**		**Pre-specialization**		**First year after specialization**		**Second year after specialization**		**Pre-specialization**		**First year after specialization**		**Second year after specialization**	
**Izumisano city**																		
Izumisano City Hospital	310	(48)	467	(55)	525	(55)	39	(39)	59	(48)	90	(56)	16	(39)	27	(37)	50	(50)
Taniguchi Hospital	469	(45)	512	(52)	537	(50)	30	(45)	34	(54)	49	(55)	11	(50)	8	(42)	16	(41)
**Kumatori city**																		
No Hospital or Clinic	0	(0)	0	(0)	0	(0)	0	(0)	0	(0)	0	(0)	0	(0)	0	(0)	0	(0)
**Kaisuka city**																		
Kaisuka City Hospital	272	(51)	0	(0)	0	(0)	29	(0)	0	(0)	0	(0)	15	(0)	0	(0)	0	(0)
Osaki Ladies’ Clinic	271	(55)	259	(51)	282	(55)	21	(57)	13	(52)	20	(51)	7	(70)	4	(33)	8	(44)
Hisamatsu Hospital	32	(7)	64	(12)	52	(11)	1	(4)	2	(7)	5	(20)	0	(0)	1	(6)	1	(10)
**North of Kishiwada city**																		
Fuchu Hospital	21	(10)	22	(8)	27	(9)	3	(13)	3	(12)	2	(7)	1	(13)	0	(0)	0	(0)
Urakawa Women’s Clinic	11	(6)	18	(8)	27	(9)	3	(13)	3	(12)	2	(7)	1	(13)	0	(0)	0	(0)
Maternal and Child Health, Osaka Medical Center	32	(22)	41	(23)	32	(19)	11	(26)	14	(23)	11	(19)	10	(29)	9	(19)	8	(19)
Oigi Ladies’ Clinic	3	(3)	12	(10)	5	(5)	0	(0)	1	(14)	1	(10)	0	(0)	0	(0)	0	(0)
Wada Tokushukai Coast Hospital	7	(8)	1	(1)	9	(8)	0	(0)	0	(0)	0	(0)	0	(0)	0	(0)	0	(0)
Izumiotsu City Hospital	11	(18)	10	(11)	6	(7)	2	(40)	2	(25)	0	(0)	1	(25)	0	(0)	0	(0)
Izumi City Hospital	2	(11)	1	(8)	0	(0)	0	(0)	0	(0)	0	(0)	0	(0)	0	(0)	0	(0)
**Southern Sennan area**																		
Kasamatsu Women’s Clinic	29	(11)	19	(8)	18	(7)	3	(18)	3	(14)	1	(5)	0	(0)	1	(20)	0	(0)
Nagamatsu Ladies’ Clinic	14	(6)	20	(8)	11	(6)	2	(11)	0	(0)	0	(0)	3	(12)	2	(10)	0	(0)
**Facilities outside the Sennan area**																		
No Answer	14	(2)	101	(12)	21	(3)	4	(4)	12	(11)	2	(3)	3	(4)	8	(11)	0	(0)
**Total**	1498		1547		1546		145		143		182		67		60		83	

*In parentheses, the percent proportions of Izumisano, Kaizuka, and Kumatori births among births in all regions were presented.

The probit regression was used in our Before and After Analysis. Before and After Analysis is often used to measure an effect of a particular treatment or event at a given period of time and entirely appropriate for such a policy evaluation that will have systematically different characteristics for all people before the policy took effect, in which the case study is of the before-and-after type. The estimation model is defined as follows:

(1) $$\begin{array}{c} Y_{\mathrm{it}}=\alpha_{0}+\alpha_{1}\;\mathrm{RegiDu}m_{i}+\alpha_{2}\;\mathrm{YearDu}m_{t}\\ \;+\alpha_{3}\left(\mathrm{RegiDu}m_{i}\times \mathrm{YearDu}m_{t}\right)+\alpha_{4}X_{\mathrm{it}}+\mu_{\mathrm{it}} \end{array}$$

The subscript *i* refers to an individual pregnant women and the subscript *t* denotes calendar year. Y is a dummy variable that equals 1 if a specified maternity facility was selected by the pregnant woman, and 0 otherwise. The regional dummy (*RegiDum*) is defined as 1 if the child was registered in Izumisano city, Kaizuka city, or Kumatori city (hereinafter referred to as Izumisano and Kaizuka cities), and 0 otherwise.

The year dummy (*YearDum*) controlling the variation of the time effect includes two dummy variables: *FirYearDum* equals 1 if the child was born during the first year after the specialization and 0 otherwise; *SecYearDum* equals 1 if the child was born during the second year after the specialization and 0 otherwise. The interaction terms of *RegiDum*_*i*_ and *YearDum*_*t*_ (*RegiDum*_*i*_ × *YearDum*_*t*_) are used here to pick up the combined effect of the region and time. X is a variable for evaluating the effect of maternity risk on hospital choice. Two factors related to high-risk pregnancies (i.e., low birth weight and premature birth) are considered in the current study. Since a strong correlation exists between these two factors, we estimate their effects separately as models 1 and 2. Finally, due to the dependent variable being a dummy variable, we assume that the error term *μ* follows the normal distribution and estimate both models by probit regression.

In our prediction, since the specialization enables Izumisano City Hospital to provide more advanced maternity services, therefore, we expect that after the specialization, high-risk pregnancies would occur more frequently in Izumisano City Hospital and normal pregnancies would be broadly distributed between Izumisano City Hospital and other maternity facilities in the area.

## Results

### Descriptive statistics

Tables 1 and 2 present the number and proportions of total births, low birth weight and premature births registered in all regions and the three communities (Izumisano city, Kaizuka city and Kumatori city) before the specialization as well as the first and the second year after the specialization, respectively.

The main results observed from the tables are that (i) the total number of births did not substantially change in the whole area before and after the specialization, (ii) after the specialization, a large progressive increase in the total number of births was observed in Izumisano City Hospital and Fuchu Hospital, (iii) as for the total births registered in the three communities (Izumisano, Kaizuka, and Kumatori) involved in the specialization, there was an increase in the number after the specialization in Izumisano City Hospital, Taniguchi Hospital, Hisamatsu Hospital; that is, pregnant women in those three cities were unlikely to choose hospitals or clinics located outside these cities, (iv) concerning high-risk deliveries, the facility having the largest number of low birth weight and premature births after the specialization was Izumisano City Hospital, followed by Taniguchi Hospital, and (v) after the specialization, the largest number of premature births was observed again in Izumisano City Hospital, followed by Osaka Medical Center for Maternal and Child Health, and then Taniguchi Hospital.

It is worthy to emphasize that in the second year after the specialization, the net increase in births with low birth weight in Izumisano City Hospital was 61, which equaled that in Kaizuka City Hospital before the specialization. In addition, the net increase of premature births in Izumisano City Hospital was 59, which was twice larger than those occurring in Kaizuka City Hospital before the specialization. With respect to the increased proportion, it was 21.78% (resp. 60.4%) in the first (resp. the second) year after the specialization for low birth weight births and 78.05% (resp. 143.9%) in the first (resp. the second) year after the specialization, compared to those before the specialization. Furthermore, due to the reason that births with low birth weight may be also premature births, we calculated the net increases in the sum of either low birth weight births or premature births. We found that the net increase (resp. the increased proportion) for this measure in Izumisano City Hospital was 36 (resp. 34.62%) in the first year after the specialization and 72 (resp. 69.23%) in the second year after the specialization, compared to those before the specialization. With respect to the ratio of high-risk births (either low birth weight births or premature births) in Izumisano City Hospital versus those at other facilities, it was 20.56%, 29.66% and 38.60% before the specialization, the first and the second year after the specialization, respectively.

### Regression analysis results

Table 3 presents the results of Before and After Analysis of pregnant women’s choice of nine maternity facilities including five hospitals (i.e., Izumisano City Hospital, Taniguchi Hospital, Hisamatsu Hospital, Fuchu Hospital, and Osaka Medical Center for Maternal and Child Health) and four clinics (i.e., Osaka Ladies’ Clinic, Urakawa Women’s Clinic, Kasamatsu Women’s Clinic, and Nagamatsu Ladies’ Clinic). Note that the births in these facilities accounted for 67.75%, 75.98%, and 78.96% of total births in all regions of the Sennan area before the specialization, in the first and the second year after the specialization, respectively.

**Table 3:** Before and after analysis for choices of major maternity facilities (N = 15,927)

	**Izumisano City Hospital**				**Taniguchi Hospital**				**Osaki Ladies’ Clinic**			
	**Model 1**		**Model 2**		**Model 1**		**Model 2**		**Model 1**		**Model 2**	
	**Coefficient marginal effect**		**Coefficient marginal effect**		**Coefficient marginal effect**		**Coefficient marginal effect**		**Coefficient marginal effect**		**Coefficient marginal effect**	
*RegiDum*	0.4003	0.0967***	0.3997	0.0967***	0.3959	0.1124***	0.3951	0.1120***	0.5367	0.0976***	0.5361	0.0973***
*FirYearDum*	0.0815	0.0186**	0.0744	0.0170*	-0.0929	-0.0246**	-0.0895	-0.0237**	0.0827	0.0135*	0.0874	0.0143*
*SecYearDum*	0.1613	0.0374***	0.1584	0.0368***	0.0002	0.0000	0.0015	0.0004	0.0573	0.0093	0.0586	0.0095
RegiDum*FirYearDum	0.2035	0.0497***	0.2110	0.0518***	0.1436	0.0405**	0.1380	0.0388**	-0.1303	-0.0196*	-0.1387	-0.0203**
RegiDum*SecYearDum	0.2306	0.0570***	0.2380	0.0591***	0.1156	0.0323**	0.1105	0.0308*	-0.0308	-0.0049	-0.0346	-0.0054
*Low Birth Weight*	0.3620	0.0941***			-0.2793	-0.0676***			-0.2568	-0.0359***		
*Premature Birth*			0.3851	0.1028***			-0.4856	-0.1052***			-0.3898	-0.0492***
*Constant*	-1.3674	***	-1.3479	***	-09899	***	-0.9937	***	-1.5297	***	-1.5361	***
**Loglikelihood**		-6484.1119		-6499.8192		-7603.7343		-7592.2292		-4845.6652		-4843.8530
	**Hisamatsu Hospital**				**Fuchu Hospital**				**Urakawa Women’s Clinic**			
	**Model 1**		**Model 2**		**Model 1**		**Model 2**		**Model 1**		**Model 2**	
	**Coefficient marginal effect**		**Coefficient marginal effect**		**Coefficient marginal effect**		**Coefficient marginal effect**		**Coefficient marginal effect**		**Coefficient marginal effect**	
*RegiDum*	-0.8712	-0.1040***	-0.8719	-0.1045***	-0.6171	-0.0468***	-0.6182	-0.0466***	-0.8159	-0.0473***	-0.8171	-0.0474***
*FirYearDum*	0.0983	0.0143**	0.1011	0.0148***	0.1975	0.0184***	0.1994	0.0185***	0.0920	0.0066*	0.0941	0.0068*
*SecYearDum*	0.0275	0.0039	0.0269	0.0039	0.2342	0.0222***	0.2342	0.0220***	0.1407	0.0103***	0.1385	0.0101***
RegiDum*FirYearDum	0.1886	0.0297*	0.1855	0.0293*	-0.1896	-0.0147	-0.1941	-0.0149	0.0779	0.0058	0.0748	0.0055
RegiDum*SecYearDum	0.1841	0.0290*	0.1785	0.0281*	-0.1377	-0.0110	-0.1388	-0.0110	0.1029	0.0077	0.0995	0.0075
*Low Birth Weight*	-0.3402	-0.0397***			-0.0162	-0.0014			-0.3458	-0.0187***		
*Premature Birth*			-0.3677	-0.0413***			-0.2636	-0.0188***			-0.4684	-0.0223***
*Constant*	-1.1954	***	-1.2067	***	-1.6386	***	-1.6282	***	-1.6578	***	-1.6654	-0.0223***
**Loglikelihood**		-4521.1634		-6499.8192		-2998.1760		-2993.3214		-2504.8155		-2505.5051
	**Maternal and Child Health, Osaka Medical Center**				**Kasamatsu Women’s Clinic**				**Nagamatsu Ladies’ Clinic**			
	**Model 1**		**Model 2**		**Model 1**		**Model 2**		**Model 1**		**Model 2**	
	**Coefficient marginal effect**		**Coefficient marginal effect**		**Coefficient marginal effect**		**Coefficient marginal effect**		**Coefficient marginal effect**		**Coefficient marginal effect**	
*RegiDum*	-0.2376	-0.0133***	-0.2353	-0.0132***	-0.6101	-0.0445***	-0.6160	-0.0439***	-0.8996	-0.0551***	-0.9018	-0.0551***
*FirYearDum*	0.0921	0.0057	0.0791	0.0049	-0.0059	-0.0005	-0.0034	-0.0003	0.0252	0.0019	0.0245	0.0018
*SecYearDum*	0.0990	0.0061*	0.1039	0.0065*	0.0212	0.0018	0.0199	0.0017	-0.1538	-0.0109***	-0.1528	-0.0108***
RegiDum*FirYearDum	0.0057	0.0003	0.0345	0.0021	-0.1685	-0.0127	-0.1697	-0.0125	0.0966	0.0077	0.1014	0.0081
RegiDum*SecYearDum	-0.1193	-0.0065	-0.1028	-0.0057	-0.2074	-0.0152*	-0.2067	-0.0148*	0.0653	0.0051	0.0673	0.0052
*Low Birth Weight*	0.7383	0.0783***			-0.1144	-0.0089*			-0.0753	-0.0053		
*Premature Birth*			0.9076	0.1157***			-0.6206	-0.0318***			0.1398	0.0116*
*Constant*	-1.9931	***	-1.9677	***	-1.5098	***	-1.4981	***	-1.5021	***	-1.5180	***
**Loglikelihood**		-2051.1838		-2044.3872		-2902.2232		-2885.7033		-2689.6015		-2688.5433

Note: ***,**, and * denote significant at 1%, 5%, and 10% levels, respectively.

In Izumisano City Hospital, the effect of *RegiDum* was significantly estimated at 0.0967 in both models, which indicated that the probability of pregnant women in Izumisano and Kaizuka cities choosing Izumisano City Hospital for their deliveries would be 9.67% higher than those living in other cities or towns. First and second year dummies (*FirYearDum* and *SecYearDum*) were significantly estimated with positive signs, after the specialization, implying that pregnant women in Sennan might choose Izumisano City Hospital. Moreover, the probability of choosing Izumisano City Hospital was greater in the second year than in the first year after the specialization, since the marginal effect of *SecYearDum* (0.0374 in model 1 and 0.0368 in model 2) was larger than that of *FirYearDum* (0.0186 in model 1 and 0.0170 in model 2) in both models. The same evidence can also be found for pregnant women living in Izumisano and Kaizuka cities (see the results of RegiDum*FirYearDum and RegiDum*SecYearDum). However, the difference between the marginal effects of the first and second year dummies was smaller in pregnant women of Izumisano and Kaizuka cities (0.0570 - 0.0497 = 0.0073 in model 1 and 0.0591 - 0.0518 = 0.0073 in model 2) than those of the whole area (0.0374 - 0.0186 = 0.0188 in model 1 and 0.0368 - 0.0170 = 0.0198 in model 2), implying that pregnant women in other cities or towns have an increasing probability of selecting Izumisano City Hospital. In addition, the marginal effects of low birth weight and premature births were significantly estimated with positive signs, which indicated that the probabilities of pregnant women facing low birth weight and premature births choosing Izumisano City Hospital were 9.41% and 10.28% higher than those with low risk births.

As for the choice of other three facilities in Izumisano and Kaizuka cities (i.e., Taniguchi Hospital, Osaki Ladies’ Clinic, and Hisamatsu Hospital) by pregnant women residing in Izumisano and Kaizuka cities, the effects of the regional dummy on the choice of Taniguchi Hospital and Osaki Ladies’ Clinic were similar to that of their choosing Izumisano City Hospital. However, the marginal effects of the first and second year dummies were quite different from those of Izumisano City Hospital. *FirYearDum* was significantly estimated to be positive in Osaki Ladies’ Clinic and Hisamatsu Hospital, and to be negative in Taniguchi Hospital. The marginal effect of *SecYearDum* was not statistically significant in any facility. The results of interaction terms between regional as well as first and second year dummies were also mixed. For Taniguchi Hospital, and Hisamatsu Hospital, similar to Izumisano City Hospital, the marginal effects of RegiDum*FirYearDum and RegiDum*SecYearDum were significantly estimated with positive signs. However, the positive propensity for choosing Taniguchi Hospital and Hisamatsu Hospital slightly lowered in the second year after the specialization. On the other hand, the marginal effects of high-risk deliveries were significantly estimated with negative signs in these three facilities, indicating that women with risky pregnancies did not choose them.

As for other facilities (i.e., Fuchu Hospital, Kasamatsu Women’s Clinic, Osaka Medical Center for Maternal and Child Health, Urakawa Women’s Clinic, and Nagamatsu Ladies’ Clinic) outside the Izumosano and Kaizuka cities, a significant negative regional effect was found in all five facilities. In Fuchu Hospital and Urakawa Ladies’ Clinic, the marginal effects of *FirYearDum* and *SecYearDum* were significantly estimated with positive signs in both models, and the marginal effects of *SecYearDum* were slightly larger than those of *FirYearDum*. With respect to the interaction terms between regional as well as first and second year dummies, the marginal effects of RegiDum*FirYearDum and RegiDum*SecYearDum were insignificant in almost all the facilities.

## Discussion

Based on the above results obtained from our empirical analysis, we discuss the research questions mentioned in the Background. With regard to the first question (Was the specialization undertaken by these gynecology and obstetrics departments a valid approach to improve the regional provision of obstetrical service in the Sennan area?), we found that after the specialization, women with risky pregnancies tended to choose Izumisano City Hospital. From the descriptive statistics, in the second year after the specialization, the net increase in low birth weight in Izumisano City Hospital was equal to that in Kaizuka City Hospital before the specialization. In addition, the net increase of premature births in Izumisano City Hospital was twice the size of that in Kaizuka City Hospital before the specialization. It should be noted that part of the increase in these high-risk births at Izumisano City Hospital may be due to it being one of a smaller number of available options however this amount cannot be quantified. Furthermore, the ratio of high-risk births (either low birth weight births or premature births) at Izumisano City Hospital versus those at other facilities was increased from 20.56% to 38.60%, which exhibited an increased tendency for high-risk births shifting from other facilities to Izumisano City Hospital. These results suggest that, to some extent, the specialization enhanced the provision of advanced obstetrical services. In our view, it is conceivable that this result was induced by the improved working environment of the obstetricians in Izumisano City Hospital. As mentioned in [12], after the gynecologists of Kaizuka City Hospital joined to work with the obstetricians at Izumisano City Hospital, the number of on-duty obstetricians at Izumisano City Hospital increased from one to two. In addition, the number of times that these employees were on duty was reduced from 5.8 times per month before the specialization to 5.1 times per month after the specialization. Moreover, the average overtime for the full-time (resp. part-time) physicians was reduced from 45.6 (resp. 76.4) hours per month before the specialization to 43.4 (resp. 70.9) hours per month after the specialization. Furthermore, the frequency of emergencies for the full-time (resp. part-time) physicians reduced sharply from 1.8 (resp. 1.6) times per month before the specialization to 0.5 (resp. 1.0) times per month after the specialization.

Concerning the second question (Did the specialization of the gynecology and obstetrics departments in Kaizuka City Hospital and Izumisano City Hospital affect pregnant women’s choices?), based on the regression analysis, we found that in addition to Izumisano City Hospital, the pregnant women with normal deliveries tended to choose the maternity facilities close to Kaizuka City Hospital but not other facilities that were far from Kaizuka City Hospital. This result is plausible because the distance from one’s residence to the maternity facility is normally an important factor considered by pregnant women when they choose where to deliver.

Finally, we note the primary limitation of our study. Although our data is unique in the existing literature, there is a lack of information about other factors that probably affect pregnant women’s choice of maternity facilities. However, as outlined in [1], the following factors significantly influence pregnant women’s choices: the cost of giving birth, the travel time to the facility, the waiting period for a medical examination, and the number of obstetricians. Therefore, we plan to include these factors in our future research. In addition, there is also a lack of information about the patient (pregnant women and/or newborns) outcomes in our data. From a policy evaluation perspective, it is extremely important to compare these outcomes before and after the specialization when evaluating whether the specialization scheme was successful or not. Due to the difficulty to obtain the patient outcome data, we leave this issue open and welcome any efforts to further explore this issue at much deeper extent.

## Conclusion

The specialization in gynecology and obstetrics departments was carried out to solve the problems of obstetrics services. This paper analyzed the effect of the specialization of the gynecology and obstetrics departments by examining the birth certificate data of 15,927 pregnant women to gain information on their choice of maternity facilities. Our results indicated that this specialization scheme was, to some extent, successful on the basis of providing maternity services for high-risk pregnancies at the prenatal care center (i.e., Izumisano City Hospital) and having created a positive effect by pregnant women to other facilities in the nearby area.

## Competing interests

The authors declare that they have no competing interests.

## Authors’ contributions

All the authors participated in the conception and design of this study. YA conducted the survey. YA, JS and KB participated in the analysis and interpretation of data. YA and JS participated in the drafting of the manuscript. All the authors read and approved the final manuscript.
